# Loss of Neurofascin-186 Disrupts Alignment of AnkyrinG Relative to Its Binding Partners in the Axon Initial Segment

**DOI:** 10.3389/fncel.2019.00001

**Published:** 2019-01-22

**Authors:** Scott A. Alpizar, Arielle L. Baker, Allan T. Gulledge, Michael B. Hoppa

**Affiliations:** ^1^Department of Biological Sciences, Dartmouth College, Hanover, NH, United States; ^2^Department of Molecular and Systems Biology, Geisel School of Medicine at Dartmouth College, Hanover, NH, United States

**Keywords:** ankyrin G, axon initial segment, voltage gated sodium channels, neurofascin-186, cultured hippocampal neurons

## Abstract

The axon initial segment (AIS) is a specialized region within the proximal portion of the axon that initiates action potentials thanks in large part to an enrichment of sodium channels. The scaffolding protein ankyrinG (AnkG) is essential for the recruitment of sodium channels as well as several other intracellular and extracellular proteins to the AIS. In the present study, we explore the role of the cell adhesion molecule (CAM) neurofascin-186 (NF-186) in arranging the individual molecular components of the AIS in cultured rat hippocampal neurons. Using a CRISPR depletion strategy to ablate NF expression, we found that the loss of NF selectively perturbed AnkG accumulation and its relative proximal distribution within the AIS. We found that the overexpression of sodium channels could restore AnkG accumulation, but not its altered distribution within the AIS without NF present. We go on to show that although the loss of NF altered AnkG distribution, sodium channel function within the AIS remained normal. Taken together, these results demonstrate that the regulation of AnkG and sodium channel accumulation within the AIS can occur independently of one another, potentially mediated by other binding partners such as NF.

## Introduction

Neurons are the most polarized electrically excitable cells, allowing for the rapid transfer of information throughout the nervous system. Postsynaptic electrical currents are received primarily by the dendrites and cell bodies, while chemical neurotransmitters are predominantly released from presynaptic boutons in the axon. The proximal region of the axon, known as the axon initial segment (AIS), maintains the molecular underpinnings of polarity (for reviews see Rasband, 2010; Huang and Rasband, 2016; Leterrier, 2018) and functionally links electrical inputs with chemical outputs through the generation of action potentials (for reviews see Clark et al., 2009; Bender and Trussell, 2012; Kole and Stuart, 2012). Action potential generation at the AIS relies on the local enrichment of a high density of voltage-gated sodium channels (Na_v_), whose arrangement within the AIS directly influences cellular excitability (Kuba et al., 2010; Grubb et al., 2011; Gulledge and Bravo, 2016). A second important factor that modulates firing of the action potential is the isolation of the AIS from the somatodendritic compartment, which generates a capacitive and conductive load that acts as a Na^+^ current sink and inhibits excitation (Brette, 2013; Eyal et al., 2014). The degree of this Na^+^ current sink is influenced by the morphology of the somato-dendritic compartment (Gulledge and Bravo, 2016; Hamada et al., 2016; Jamann et al., 2018; Kole and Brette, 2018). Taken together, factors controlling the location and function of Na_v_ within the AIS are critical influences on excitability, though this influence will vary across cell types due to their respective morphology.

The molecular development of the AIS is led by the scaffolding protein ankyrinG (AnkG), which has been dubbed the “master regulator” of the AIS (Jenkins and Bennett, 2001; Rasband, 2010; Grubb et al., 2011; Leterrier et al., 2015). AnkG typically arrives during the first few days of development, at about the same time that a single neurite adapts an axon-like extension (Boiko et al., 2007; Hedstrom et al., 2007; Galiano et al., 2012; Le Bras et al., 2014; Kyung et al., 2017). Unsurprisingly, due to its early arrival during axon extension, AnkG is thought to directly influence the subsequent arrival of Na_v_, with various Na_v_ isoforms arriving throughout AIS maturation, including Na_v_1.6 (Boiko et al., 2007; Hedstrom et al., 2007). A number of other proteins containing AnkG binding domains also enrich during this time period, including the cell adhesion molecules (CAMs) neurofascin-186 (NF-186) and neuronal CAM (NrCAM; Hedstrom et al., 2007). Genetic ablation of AnkG after AIS formation causes the dispersion of other AIS proteins, including Na_v_ and NF (Hedstrom et al., 2008). Additionally, mutations in the AnkG binding site of Na_v_ disrupt its targeting and accumulation at the AIS (Gasser et al., 2012). Indeed, when exogenously expressed, the fragment of the Na_v_ containing this targeting motif can enrich within the AIS (Garrido et al., 2001) through a process involving homogenous delivery to the somatic membrane and selective endocytic elimination from areas outside the AIS (Fache et al., 2004). However, these results do not eliminate other mechanisms that may be controlling enrichment. Addition of an AnkG binding motif to other ion channels does not cause enrichment of these channels at the AIS, suggesting this motif alone is not solely regulating the delivery of Na_v_ to the AIS for enrichment (Akin et al., 2015). Furthermore, Na_v_ often exist in neurons as heteromeric trimers with a single alpha (α) subunit and two transmembrane beta (β) subunits, which add additional layers of interactions to their trafficking (Catterall, 2000). The knockout of the Na_v_ β1 subunit in mice results in a failure to accumulate Na_v_1.6 at the AIS, but instead leads to elevated levels of Na_v_1.1 accumulation as a compensatory mechanism (Brackenbury et al., 2010). The β1 subunit also contains its own AnkG binding motif (Malhotra et al., 2002) and, along with β3 subunits, interacts with NF through extracellular immunoglobulin (Ig) domains (Ratcliffe et al., 2001). These interactions suggest that the coupling and alignment of AnkG and Na_v_ within the mature AIS may experience additional regulation outside of their direct interaction.

NF has been implicated in cellular functions outside of the AIS. For instance, NF null mice die by postnatal day 7 (P7) and fail to recruit Na_v_ to CNS nodes of Ranvier (Sherman et al., 2005). In addition, while NF null mice show a normal initial recruitment of Na_v_ to the AIS in cerebellar Purkinje cells, this is followed by a complete loss of both Na_v_ and AnkG after 15 days (Zonta et al., 2011). Knocking down NF in hippocampal cultures also leads to an impairment in AnkG enrichment (Leterrier et al., 2017). Thus, AnkG is sensitive to the overall stability of interacting partners at the AIS. Therefore, while NF may be influencing the biophysical properties of Na_v_ directly through preferential recruitment or retention of specific β subunits, these data also suggest that NF may influence Na_v_ accumulation and localization indirectly through its interaction with AnkG. To further clarify the role of NF at the AIS during maturation, we measured parameters of AIS development, composition, and cellular function in wild-type and genetically manipulated neurons. Utilizing the CRISPR/Cas9 system, we targeted a single guide RNA (sgRNA) to NF in cultured hippocampal neurons shortly after initial AIS development. Neurons expressing NF sgRNA exhibited a loss of AnkG enrichment as well as a distal shift in the AnkG localization independent of Na_v_ within the AIS, suggesting a previously unreported and selective role for NF in the localization and enrichment of AnkG within the mature AIS.

## Materials and Methods

### Animals

This study was carried out in accordance with the recommendations of Dartmouth College’s Institutional Animal Care and Use Committee (IACUC). The protocol was approved by Dartmouth College’s Institutional Animal Care and Use Committee—Protocol 00002115.

### Cell Culture

Neurons from the hippocampal CA1–CA3 regions were dissected from P1 Sprague-Dawley rats, dissociated (bovine pancreas trypsin; 5 min at room temperature), and plated on polyornithine-coated coverslips inside a 6 mm diameter cloning cylinder as previously described (Hoppa et al., 2012). Calcium phosphate mediated transfection was performed on 5-day-old cultured neurons with the described plasmids (below).

### Antibodies and Plasmids

Mouse monoclonal antibodies to AnkG (1:1,000, 75-187 NeuroMab), panNa_v_ (1:1,000, S8809 Sigma), and NF (1:1,000, 75-172 NeuroMab for external; 1:1,000, 75-027 NeuroMab for internal), rabbit polyclonal antibodies to AnkG (1:500, 386-003 Synaptic Systems), NrCAM (1:1,000, ab24344 Abcam) and mCherry (1:2,000, ab167453 Abcam), a chicken polyclonal antibody to GFP (1:2,000, A10262 Thermo Fisher, Waltham, MA, USA), a guinea pig polyclonal antibody to MAP2 (1:2,000, 188-004 Synaptic Systems), and a 565-FluoTag camelid monoclonal antibody to RFP (1:250, N0404-At565-S Synaptic Systems) were used (Grubb and Burrone, 2010a; Xu et al., 2013; Leterrier et al., 2017; Lezmy et al., 2017). Alexa Fluor 488-, 546-, and 647-conjugated goat anti-rabbit, anti-mouse, and anti-chicken IgG (Cat. #s A11034, A11039, A11029, A11074, A11035, A21236, A21245) were used for secondary staining (1:1,000, Thermo Fisher, Waltham, MA, USA).

To construct the NF sgRNA, we inserted a guide RNA (sgRNA) targeting NF-186 specifically (using the sequence caccgTCAACATTGCCAAGGACCCA for the forward primer and GTCAACATTGCCAAGGACCCAgttt for the reverse primer) into the pU6-(BbsI)CBh-Cas9-T2A-mCherry plasmid purchased from Addgene (plasmid 64324). The empty pU6-(BbsI) CBh-Cas9-T2A-mCherry plasmid was used as the sgRNA control. Na_v_1.6-GFP was cloned as previously described (Gasser et al., 2012).

### Immunofluorescence

To visualize AIS proteins, days *in vitro* (DIV) 14–17 neurons were fixed with 4% paraformaldehyde and 4% sucrose in phosphate buffered saline (PBS) and permeabilized with 10% Triton X-100 and 10% goat serum in PBS for 30 min, a procedure to help visualize Na_v_ localization at the AIS (Akin et al., 2015). Neurons were then incubated with the appropriate primary antibodies overnight (~16 h) and visualized using Alexa Fluor-conjugated secondary antibodies, both in 5% goat serum.

### Image Acquisition

Images of stained neurons were primarily obtained using an Olympus microscope (IX-83) equipped with a 40× 1.35 NA oil immersion objective (UAPON40XO340-2). Illumination was generated with a halogen light source (X-Cite 120PC Q; Excelitas) and images captured with an IXON Ultra 897 EMCCD camera (Andor). Green fluorescence was captured using filter sets including ET470/40×, ET525/50m, and T495lpxr filters; red fluorescence was captured using filter sets including ET560/40×, ET630/75m, and T585lpxr filters; and far-red fluorescence was captured using filter sets including ZET635/20×, ET655lpm, and ZT640rdc filters (all from Chroma). All images were captured as a time series of 15 brief exposures which were then maximum intensity projected for analysis. In order to eliminate increased levels of background in the staining, z-stacks were obtained for NrCAM stained neurons using confocal imaging on a Zeiss LSM 880 microscope with a 40×, 1.3 NA objective. Z-stacks contained a step size of 0.35 μm and ranged from 3 to 6 μm in height to ensure all AIS signal was captured. For MATLAB intensity profiling (see below), channels were merged together to create an RGB image using Fiji^1^.

### Electrophysiology

Neurons were cultured and grown as indicated above for 14–18 days before being transferred to a recording chamber for electrophysiological recording. Neurons were continuously perfused (at 35–36°C) with oxygenated artificial cerebrospinal fluid composed of the following (in mM): 125 NaCl, 25 NaHCO_3_, 3 KCl, 1.25 NaH_2_PO_4_, 2 CaCl_2_, 1 MgCl_2_, and 25 glucose (saturated with 95% O_2_–5% CO_2_). Neurons were visualized with a 60x water immersion objective on an Olympus BX51WI microscope. Whole-cell current-clamp recordings of neurons were made with patch pipettes (5–7 MΩ) filled with a standard intracellular solution containing (in mM): 135 K-gluconate, 2 NaCl, 2 MgCl_2_, 10 HEPES, 3 Na_2_ATP, and 0.3 NaGTP (pH 7.2 with KOH). Wide-field epifluorescence illumination using a 550 nm LED was used to identify transfected neurons for whole-cell recording. Data were acquired with Axograph software (Axograph Scientific) using BVC-700 amplifiers (Dagan Corporation) and ITC-18 digitizers (HEKA Instruments). Membrane potentials were sampled at 25 kHz, filtered at 5 kHz, and corrected for the junction potential of +12 mV. Capacitance was maximally compensated and bridge-balance used to compensate for series resistance [~10–25 MΩ, which was stable (within ±5 MΩ) throughout experiments] as previously described (Gulledge et al., 2009). Cells that showed large changes in series resistance were discarded for data analysis purposes. Depolarizing current injections were titrated to evoke just-suprathreshold action potentials and measurements were made of spike threshold as well as the peak, rise time, width, and decay time of the action potential waveform. Input resistance was calculated from the slope of the linear portion of the steady-state voltage-current relationship established with a sequence of somatic current injections (usually −50 to +50 pA). All analyses of action potentials were made from 10 or more trials of the stimulus protocol. Action potential threshold was defined as the voltage at the time corresponding to the slope exceeding 50 mV/ms. Action potential amplitudes were measured as the absolute peak positive amplitude of the voltage response following the current step, relative to the membrane potential occurring just before the initiation of the action potential. Action potential rise time was calculated as time from 10 to 90% of the peak. Full width at half maximum amplitude (FWHM) refers to the broadness of the action potential measured at 50% of peak amplitude. Decay time was calculated as time from 100 to 50% of the peak.

### Image and Data Analysis

For intensity measurements, images were analyzed in Fiji^1^. A 2-pixel wide line was drawn from the soma to the distal axon to a minimum length of ~40 μm. The start of the AIS was identified by the morphological constriction of the soma. In cases where an AIS was found on a proximal dendrite, the constriction of the dendrite was used in the same manner as if it were the soma. The raw fluorescence values were then copied into a custom-written Excel spreadsheet to define the AIS (continuous normalized fluorescence intensity above 0.33 for more than 5 μm; termination of the AIS was identified if the normalized fluorescence intensity dropped below 0.33 for more than 2 μm). This region was averaged, and the background was subtracted. Intensity values are represented as the ratio of fluorescence intensity of transfected neurons to the fluorescence intensity of multiple untransfected neurons in the same image (Leterrier et al., 2017). For determination of AIS distance from the soma and length, MATLAB software was used^2^. A previously published MATLAB code (Grubb and Burrone, 2010a) downloaded from the Grubb Lab was used to obtain raw 3 × 3 pixel measurements that were normalized and adjusted in a custom-written Excel spreadsheet to obtain length and distance from the soma of the AIS. AIS localization index was calculated for each antibody from untransfected cells using the following formula:


AIS Localization Index = (MeanAIS−Meannon-AIS)/(MeanAIS+Meannon-AIS)

where MeanAIS is the mean fluorescence within the AIS region as defined above and Meannon-AIS is the mean fluorescence over all points outside of the AIS as previously described (Grubb and Burrone, 2010b). All measurements of enrichment at the AIS comparing AnkG and Na_v_ localization in both sgRNA control and NF sgRNA conditions were independently replicated blind to experimental conditions. All physiological data were analyzed using Axograph software.

### Statistical Analysis

All data are presented as mean ± standard error of the mean (SEM). Significance was calculated for two conditions using two-tailed Student’s *t*-test, except in paired distance from the soma measurements, where a Wilcoxon signed-rank test was used. For three or more conditions, a one-way ANOVA followed by Tukey’s multiple comparison analysis was performed, except for any distance from the soma measurements, where a Kruskal-Wallis ANOVA followed by a Dunn’s post-test with Bonferroni correction was performed. In all figures significance is indicated as: *p < 0.05; **p < 0.01; ***p < 0.001. All statistical tests were performed using OriginPro 8 or R software.

## Results

### AnkG and NF Arrive at the AIS Prior to Na_v_

**Figure 1:**
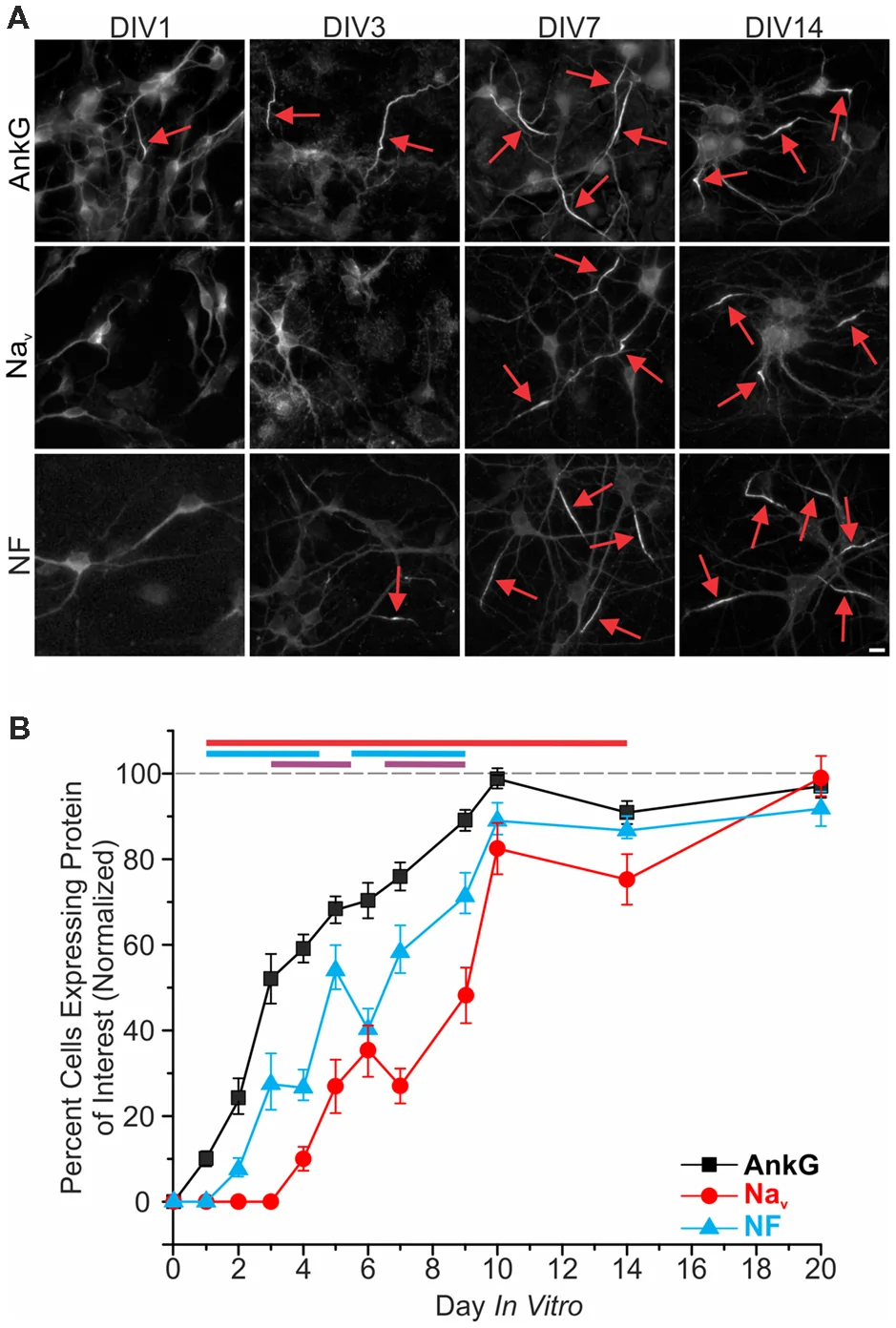
Development of the axon initial segment (AIS). **(A)** Representative images of immunostaining for ankyrinG (AnkG), panNa_v_, and neurofascin (NF) at days *in vitro* (DIV)1, 3, 7, and 14. Red arrows indicate AIS enrichment. Scale bar: 10 μm. **(B)** Percentages of neurons containing discernable enrichment at the AIS compared to the soma for AnkG (black), Na_v_ (red), and NF (cyan) during development (≥10 images/fields of view were quantified for all proteins in all DIV). Error bars indicate mean ± standard error of the mean (SEM). Cyan line at top indicates statistical significance between NF and AnkG, red line indicates statistical significance between Na_v_ and AnkG, and purple line indicates that Na_v_ is statistically significant from NF. All significance indicates *p* < 0.05, ANOVA with Tukey’s *post hoc* comparisons.

One of the earliest proteins to localize to the proximal axon during the establishment of the AIS is AnkG, as previously demonstrated both *in vitro* (Boiko et al., 2007; Hedstrom et al., 2007) and *in vivo* (Galiano et al., 2012; Le Bras et al., 2014). We sought to determine the time course of AIS enrichment for NF and Na_v_, which both contain different AnkG binding domains. Hippocampal neurons from P1 rat pups were dissociated, plated, and then fixed at specific 24-h time points until DIV20. At each time point, we evaluated protein enrichment using immunostaining against AnkG, panNa_v_, and NF. Accumulation of AnkG staining at the AIS was rapid, with discernable enrichment compared to the soma found in 10 ± 2% of neurons at DIV1 (*n* = 39 fields of view; an average of five cells were contained in each field of view), and appeared in a majority of neurons on DIV3 (Figures 1A,B). NF was the next observable protein to enrich within the AIS, present in over 50 ± 6% (*n* = 25 fields of view) of neurons at DIV7. Na_v_ were the last to enrich in the AIS, reaching 50 ± 6% (*n* = 18 fields of view) of neurons at DIV9, as detected by a panNa_v_ antibody in good agreement with previous findings (Yang et al., 2007). Expression of all three proteins gradually increased after their initial observations, becoming evident in greater proportions of neurons in subsequent days, with more than 75% of neurons expressing all three proteins by DIV10 (AnkG 98 ± 1%, NF 89 ± 4%, Na_v_ 82 ± 6%; *n* = 26, 11, and 15 fields of view, respectively).

**Figure 2:**
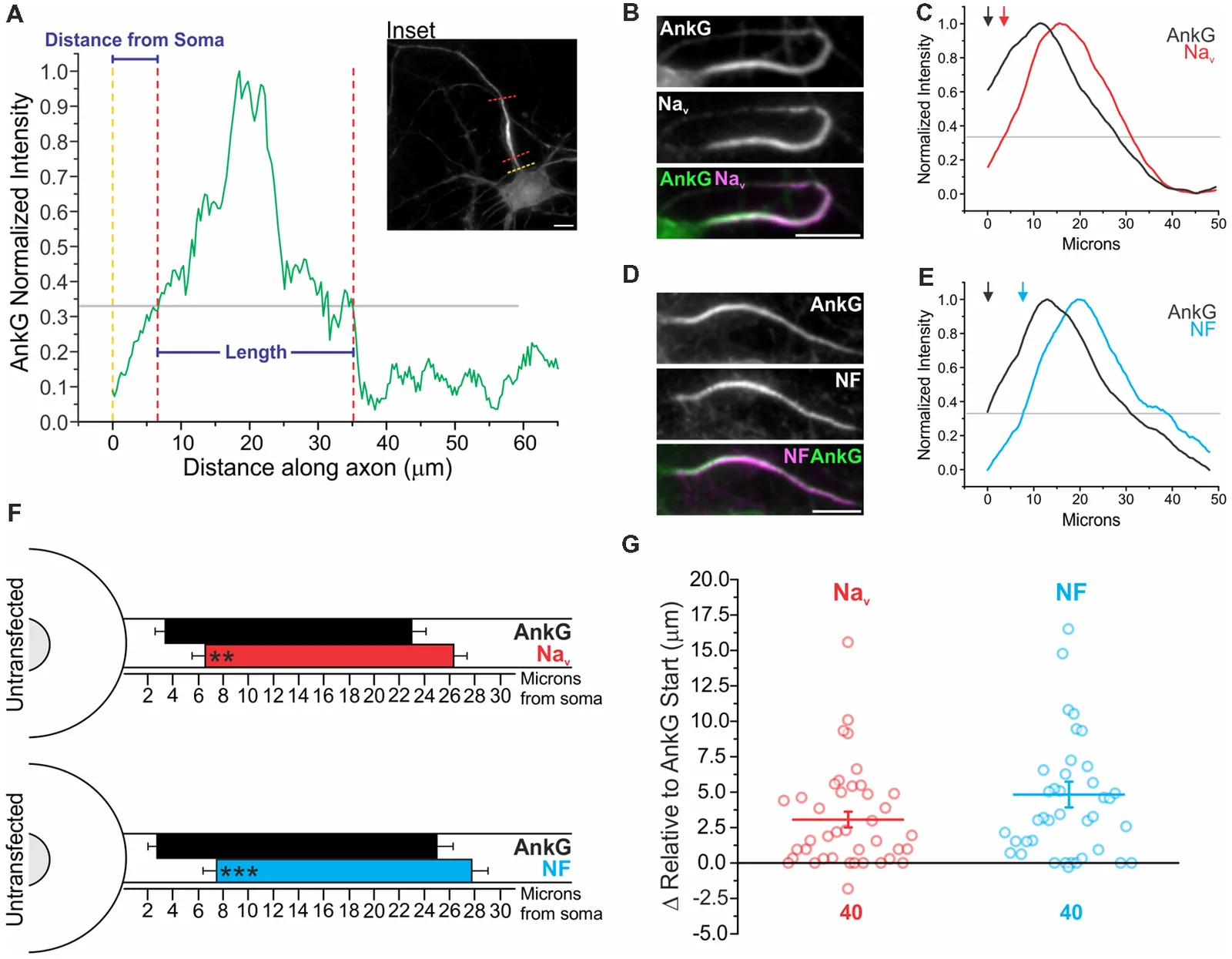
AnkG enriches proximally relative to its binding partners at the AIS. **(A)** Representative AIS intensity plot for AnkG staining along the proximal axon (see inset) indicating parameters obtained (distance from the soma and length). Gray line indicates the standard 0.33 normalized intensity threshold to define the AIS. Yellow dashed line indicates the junction of the soma and axon where intensity profiling began; red dashed lines indicate start and end parameters of the AIS found as described in the “Materials and Methods” section. Scale bar: 10 μm. **(B)** Representative images of immunostaining for AnkG with panNa_v_. AnkG and Na_v_ are pseudo-colored green and magenta in overlay for contrast. Scale bar: 10 μm. **(C)** Smoothed and normalized fluorescence intensity traces for AnkG (black) and Na_v_ (red) from the images in **(B)**. Gray line indicates fluorescence intensity threshold to define the AIS. Arrows indicate start of AnkG (black) or Na_v_ (red) enrichment above threshold. **(D)** Representative images of immunostaining for AnkG with NF. AnkG and NF are pseudo-colored green and magenta in overlay for contrast. Scale bar: 10 μm. **(E)** Smoothed and normalized fluorescence intensity traces for AnkG (black) and NF (cyan) from **(D)**. Gray line indicates intensity threshold to define the AIS. Arrows indicate start of AnkG (black) or NF (cyan) enrichment above threshold. **(F)** To-scale distribution of AnkG (black; *n* = 40) and Na_v_ (red; *n* = 40; top), or AnkG (black; *n* = 40) and NF (cyan; *n* = 40; bottom) enrichment in untransfected DIV14 neurons. Left error bar indicates SEM for distance from the soma, right error bar indicates SEM for length. Asterisks indicate significance from AnkG start; **p = 0.0035 for Na_v_ and ***p < 0.001 for NF, Wilcoxon signed-rank test. **(G)** Comparison of start of protein enrichment for Na_v_ (red) or NF (cyan) relative to AnkG start (black horizontal line) in the same cells. Error bars indicate mean ± SEM, *n* = 40 for both proteins.

To determine the relative localization of AIS proteins with respect to each other at the AIS, intensity profiles of immunostaining within the axon were obtained. A line was drawn over the AIS from the point at which the soma meets the axon and extended distally. An intensity profile plot was obtained, and a continuous portion having greater than 33% normalized intensity and longer than 5 μm was defined as the AIS as previously described (Grubb and Burrone, 2010a) and detailed in the “Materials and Methods” section (Figure 2A). Intensity profiling allowed us to obtain the distance from the soma as well as the length of AnkG, panNa_v_, and NF in untransfected DIV14 neurons (Figures 2B–G). AnkG was most often localized proximal to both Na_v_ and NF enrichment. To systematically quantify this distal localization of NF and Na_v_, we immunostained identical cells with AnkG and either NF or panNa_v_. While all three proteins exhibited enriched AIS staining of similar length, there were protein-specific differences in their relative location within the AIS. The start of Na_v_ enrichment was localized distal to that of AnkG (3.3 ± 0.7 μm and 6.4 ± 1.0 μm for AnkG and Na_v_ respectively, *n* = 40), as was the start of enrichment for NF [2.7 ± 0.76 μm and 7.5 ± 1.0 μm for AnkG and NF respectively, *n* = 40; Figure 2F. Start positions of both Na_v_ (3.0 ± 0.6 μm) and NF (4.8 ± 0.9 μm) relative to AnkG start can be seen in Figure 2G]. We used a heavy detergent (10% Triton X-100) permeabilization to optimize Na_v_ detection as previously described (Akin et al., 2015). To ensure that this did not contribute to our findings of relative enrichment, we directly compared AnkG and NF under high (10% Triton X-100) and low (0.2% Triton X-100) detergent conditions and found that the distance from the soma and length of the two proteins were not altered by permeabilization (Supplementary Figure S1). A final concern when using this quantification of AIS parameters as determined by immunostaining is comparable signal to noise. To address this, we calculated the AIS localization index (Grubb and Burrone, 2010b) for each antibody used and found that each had a high localization value that was statistically indistinguishable from the others, validating the method (Supplementary Figure S2A).

### Knockout of NF Influences AnkG Localization and Enrichment at the AIS

**Figure 3:**
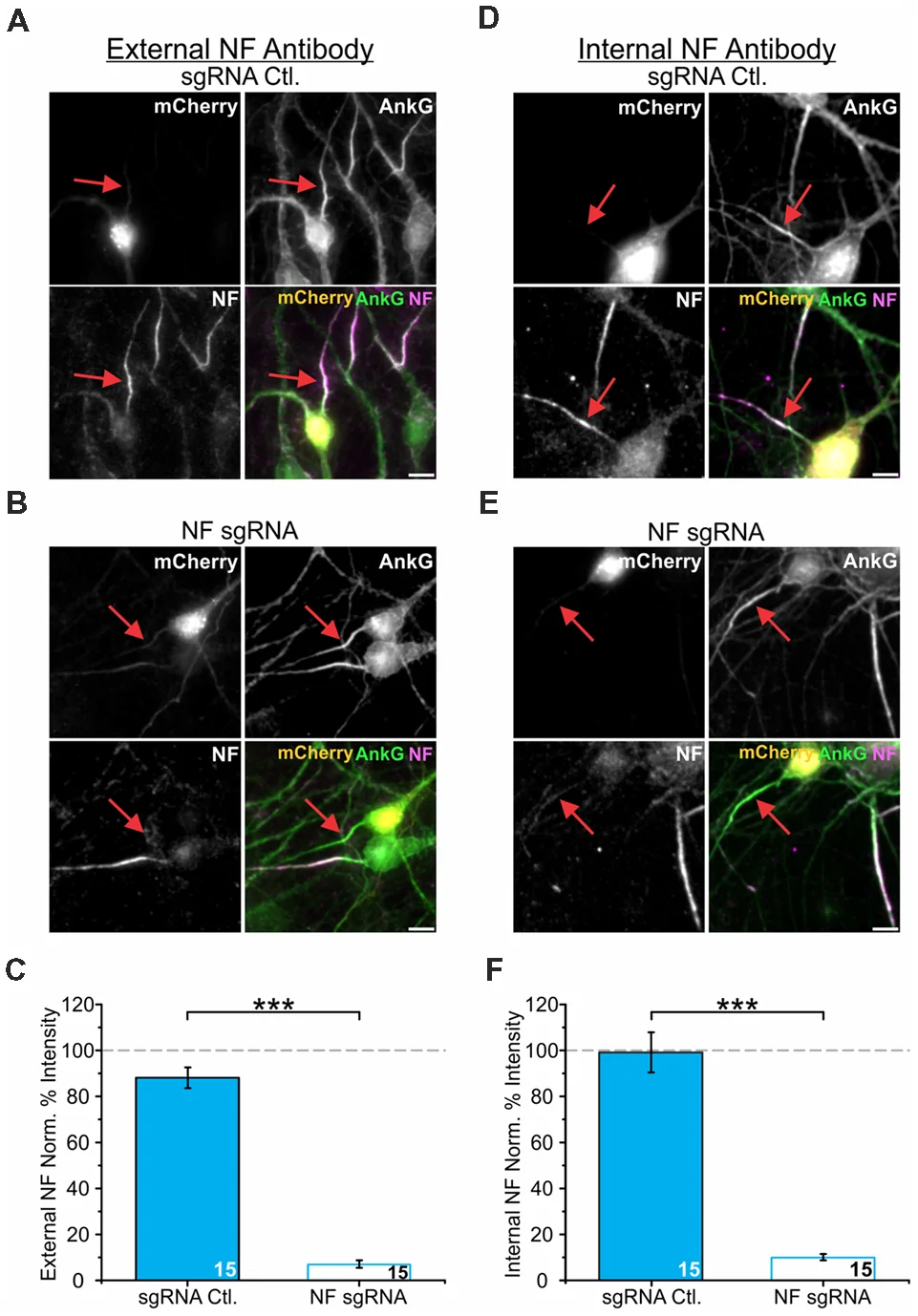
NF single guide RNA (sgRNA) successfully depletes NF enrichment in the AIS. **(A,B)** Representative images of DIV14 sgRNA control **(A)** or NF sgRNA **(B)** neurons stained for mCherry, AnkG, and NF using an antibody targeting an extracellular domain of NF. Red arrows indicate the AIS of transfected neurons. Scale bar: 10 μm. **(C)** Ratio of mean fluorescence intensity of NF at the AIS using an externally targeting antibody in sgRNA control or NF sgRNA expressing neurons, normalized to untransfected neurons in the same image (*n* = 15 for both conditions; ***p < 0.001, Student’s *t*-test). **(D,E)** Representative images of DIV14 sgRNA control **(D)** or NF sgRNA **(E)** neurons stained for mCherry, AnkG, and NF using an antibody targeting an intracellular domain of NF. Red arrows indicate the AIS of transfected neurons. Scale bar: 10 μm. **(F)** Ratio of mean fluorescence intensity for external NF at the AIS using an internally targeting antibody in sgRNA control or NF sgRNA expressing neurons, normalized to untransfected neurons in the same image (*n* = 15 for both conditions; ***p < 0.001, Student’s *t*-test). Error bars indicate mean ± SEM.

While Na_v_ α subunits have an AnkG binding domain sufficient to target them to the AIS, it has also been shown that Na_v_ accumulation stabilizes AnkG enrichment within the AIS (Leterrier et al., 2017). Moreover, Na_v_ are most commonly found in the brain as heterotrimeric complexes with one α subunit and two β subunits (Catterall, 2000; Namadurai et al., 2015). NF is uniquely positioned to bind to both AnkG intracellularly through its FIGQY motif and extracellularly with Na_v_ β subunits through its Ig domains (Ratcliffe et al., 2001). To elucidate how these interactions may be involved in the localization of Na_v_ relative to AnkG post-development, we measured enrichment levels of both AnkG and Na_v_ after acute depletion of NF from the maturing AIS. We developed a CRISPR-based method to ablate endogenous protein levels of NF. We combined this with a sparse transfection method (Ca^2+^-phosphate) that targeted ~1% of neurons for NF depletion. Recent work has demonstrated that large scale changes in electrical activity within a dish can alter AIS localization relative to the soma (Grubb and Burrone, 2010a). Thus, this combination of CRISPR and sparse transfection closely simulates knockout and avoids any potential large-scale alterations in the overall electrical activity within the culture. A single plasmid encoded the sgRNA, Cas9 enzyme, and a fluorescent protein marker of transfection (mCherry) with cDNA coexpressed using a ribosomal cleavage site as previously described (Cho et al., 2017). This provided both a means to visualize the transfected neurons as well as ensured proper CRISPR component targeting. To test the effectiveness of our sgRNA for CRISPR depletion during AIS maturation, neurons were transfected at DIV5 with either NF sgRNA or an “empty” sgRNA control plasmid that only expressed Cas9 and mCherry (sgRNA Ctl.). Neurons were then fixed and immunostained for mCherry, NF and AnkG. We verified the efficiency of our NF sgRNA construct by measuring the NF labeling intensity (Figures 3A,B). The intensity of NF labeling, as detected using an antibody directed against an extracellular domain of NF, was severely reduced in NF sgRNA neurons to 7 ± 2% (*n* = 15) of that observed in untransfected neurons in the same image. In contrast, neurons without sgRNA, but expressing Cas9 and mCherry served as our control condition and did not show significant depletion of NF (88 ± 5%, *n* = 15; Figure 3C). To ensure that the use of a CRISPR InDel for knockout did not produce a NF truncation mutant, we also probed NF levels using an antibody directed against the intracellular N-terminal domain of NF and observed similar reductions in NF protein in sgRNA transfected neurons (Figures 3D–F, **F**: 99 ± 9% for sgRNA Ctl. and 10 ± 1% for NF sgRNA, *n* = 15 for both conditions). Additionally, this intracellular NF antibody showed a similar distal localization relative to AnkG in untransfected cells (Supplementary Figures S2B,C) compared to measurements using the extracellular NF antibody (Figure 2F).

**Figure 4:**
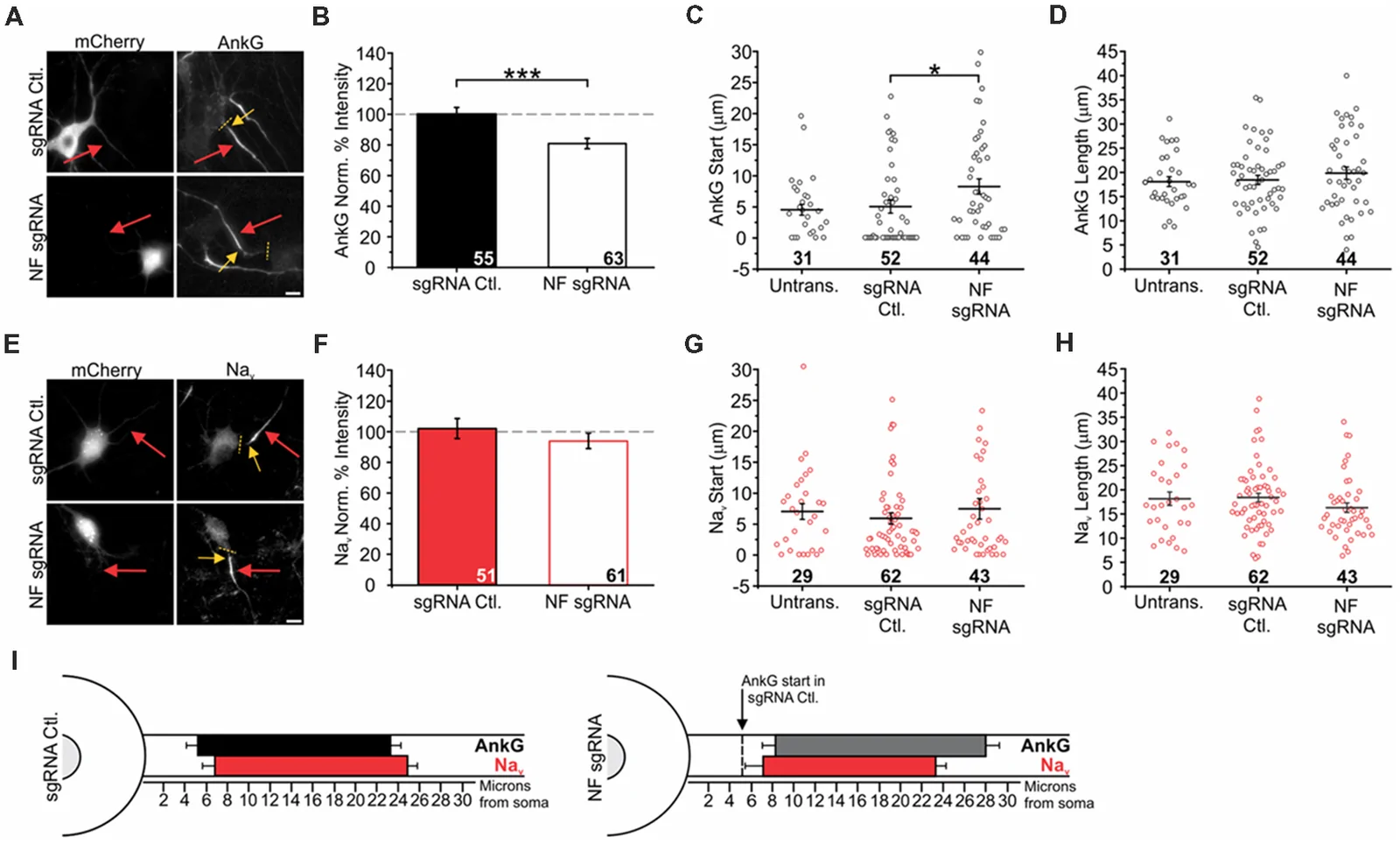
Knockout of NF disrupts AnkG enrichment and relative localization to Na_v._ **(A,E)** Representative images for sgRNA control or NF sgRNA stained for mCherry with AnkG **(A)** or panNa_v_ **(E)**. Red arrows indicate the AIS of transfected neurons; yellow arrows indicate start of enrichment; yellow dashed lines indicate junction of soma and axon. Scale bars: 10 μm. **(B,F)** Ratio of mean fluorescence intensity for AnkG **(B)** and Na_v_ **(F)** at the AIS in sgRNA control or NF sgRNA neurons normalized to adjacent untransfected neurons in the same image (**B**: sgRNA Ctl. *n* = 55; NF sgRNA *n* = 63; ***p < 0.001, Student’s *t*-test. **F**: sgRNA Ctl. *n* = 51; NF sgRNA *n* = 61). **(C,G)** Distance from the soma to start of AIS enrichment for AnkG **(C)** and Na_v_ **(G)** in untransfected, sgRNA control, and NF sgRNA neurons (**C**: Untransfected *n* = 31; sgRNA Ctl. *n* = 52; NF sgRNA *n* = 44; *p = 0.024, Kruskal-Wallis ANOVA followed by Dunn’s post-test with Bonferroni correction. **G**: Untransfected *n* = 29; sgRNA Ctl. *n* = 62; NF sgRNA *n* = 43). **(D,H)** AIS length for AnkG **(D)** and Na_v_ **(H)** in untransfected, sgRNA control and NF sgRNA neurons (**D**: Untransfected *n* = 31; sgRNA Ctl. *n* = 52; NF sgRNA *n* = 44. **H**: Untransfected *n* = 29; sgRNA Ctl. *n* = 62; NF sgRNA *n* = 43). Error bars indicate mean ± SEM.** (I)** To-scale distribution of AnkG (black) and Na_v_ (red) in sgRNA control (left) and NF sgRNA (right) neurons. Color saturation of bars are indicative of average labeling intensity of the population. Dashed black line indicates start of AnkG enrichment in sgRNA control. Left error bar indicates SEM for distance from the soma, right error bar indicates SEM for length; data taken from **(B–D)** and** (F–H)**.

Previous studies have found that NF is more important for the stabilization of AIS components than for their delivery to the AIS (Zonta et al., 2011). Given that NF has binding sites for both AnkG and Na_v_ β subunits, we wanted to determine whether our NF CRISPR knockout would destabilize the arrangement of molecular components within the AIS. In DIV14 neurons AnkG labeling intensity was significantly decreased in neurons transfected with the NF sgRNA construct compared to the sgRNA control (100 ± 4% for sgRNA Ctl., 81 ± 3% for NF sgRNA; *n* = 55 and 63, respectively; Figures 4A,B), results that are in good agreement with those of a previous study using shRNA in hippocampal neurons (Leterrier et al., 2017). We additionally undertook detailed measurements of AnkG localization within the axon using a previously developed MATLAB code (Grubb and Burrone, 2010a). First, we quantified the distance from the soma that the AIS started, as measured by AnkG staining. Interestingly, we measured a distal shift in the start point for AnkG enrichment in NF sgRNA neurons (8.3 ± 1.2 μm, *n* = 44) compared to sgRNA control (5.0 ± 1.0 μm, *n* = 52) and untransfected neurons (4.5 ± 0.9 μm, *n* = 31; Figure 4C). Despite the change in start location, the overall length of the AnkG enrichment was similar in the various conditions (18.1 ± 1.0 μm for untransfected, 18.4 ± 1.0 μm for sgRNA Ctl., 19.8 ± 1.3 μm for NF sgRNA; *n* = 31, 52, and 44, respectively; Figure 4D). Therefore, the loss of NF results in a decrease in the overall enrichment of AnkG and a distal shift in its distance from the soma.

### NF Knockout Disrupts Stereotypical Alignment of Na_v_ Relative to AnkG Within the AIS

Next, we sought to determine if the loss of NF caused a uniform shift in the enrichment and localization of Na_v_ as a result of their AnkG binding motif dictating a distal shift coupled to AnkG. Previous experiments investigating translocation of the AIS during plasticity indicate that AnkG, Na_v_, and NF move uniformly together, without noticeable changes in overall enrichment (Grubb and Burrone, 2010a). Despite a ~20% decrease in AnkG enrichment, Na_v_ enrichment at the AIS was unchanged compared to adjacent untransfected neurons (102 ± 6% for sgRNA Ctl.; 94 ± 5% for NF sgRNA; *n* = 51 and 61, respectively; Figures 4E,F). Moreover, when we measured the distance from the soma of AIS start as well as the length of the AIS in untransfected, sgRNA control, and NF sgRNA neurons using Na_v_ staining, we found that AnkG and Na_v_ were uncoupled. While the start of AnkG enrichment was distally shifted by 3.3 microns (see above), we observed no differences in either the start locations (7.0 ± 1.3 μm for untransfected, 5.9 ± 0.9 μm for sgRNA Ctl., 7.5 ± 1.7 μm for NF sgRNA; *n* = 29, 62, and 43, respectively; Figure 4G) or length (18.2 ± 1.4 μm for untransfected, 18.4 ± 0.9 μm for sgRNA Ctl., 16.3 ± 1.0 μm for NF sgRNA; *n* = 29, 62, and 43, respectively; Figure 4H) of Na_v_ enrichment. Collectively, these data demonstrate, to our knowledge, the first changes in AnkG localization that are independent of Na_v_ within the AIS, suggesting a unique role for NF to align the enrichment between AnkG and Na_v_ in the mature AIS (summary in Figure 4I).

### Overexpression of Na_v_ Restores AnkG Enrichment, but Not Relative Localization

**Figure 5:**
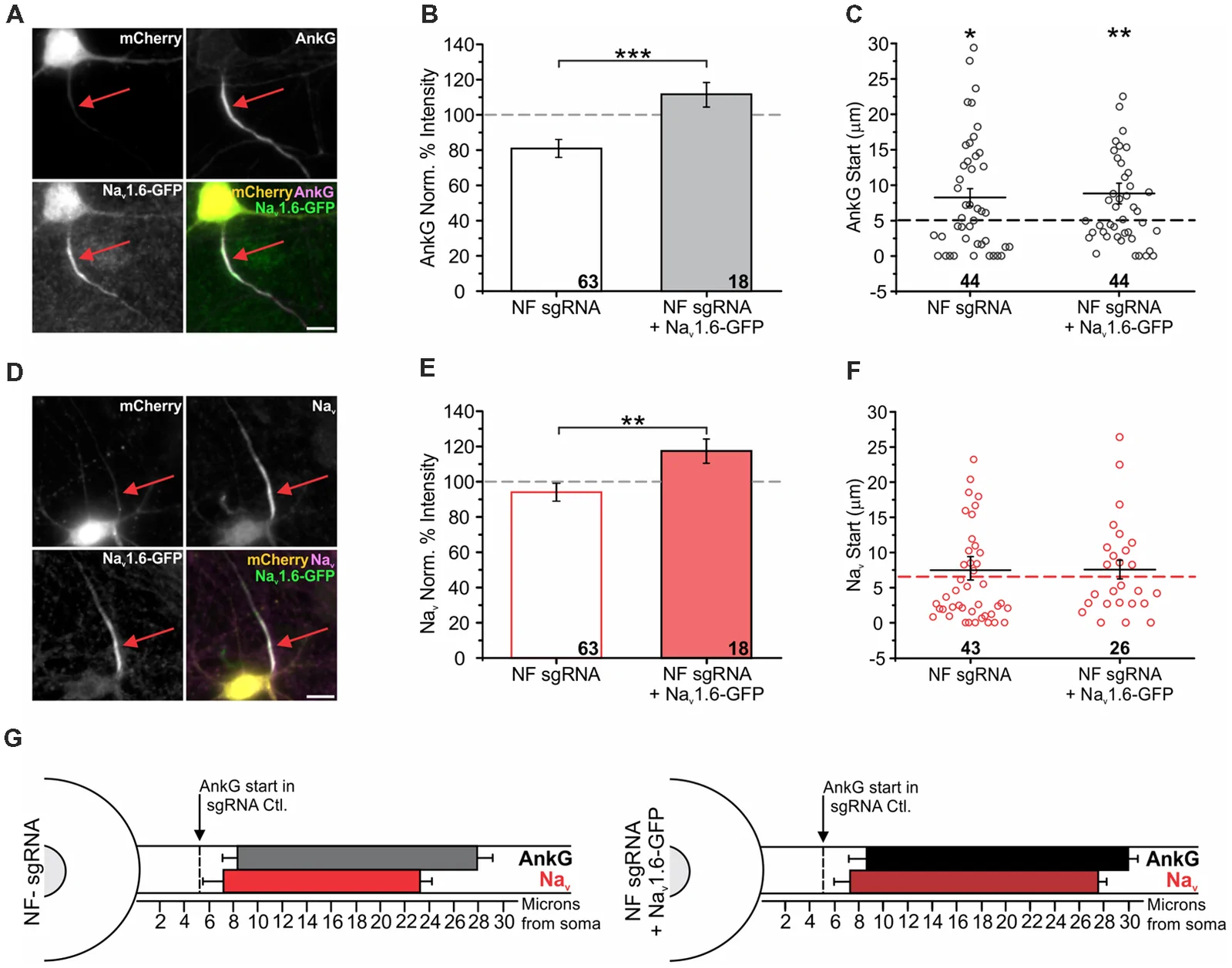
Na_v_1.6 overexpression rescues AnkG enrichment, but not translocation. **(A,D)** Representative images for NF sgRNA + Na_v_1.6-GFP co-transfected neurons stained for mCherry, GFP, and AnkG **(A)** or panNa_v_ **(D)**. Red arrows indicate the AIS of transfected neurons. Scale bars: 10 μm. **(B,E)** Ratio of mean fluorescence intensity for AnkG **(B)** and Na_v_ **(E)** at the AIS in NF sgRNA (originally from Figure 3) or NF sgRNA + Na_v_1.6-GFP expressing neurons normalized to untransfected neurons in the same image (**B**: NF sgRNA *n* = 63; NF sgRNA + Na_v_1.6-GFP *n* = 18; ***p < 0.001, Student’s *t*-test. **E**: NF sgRNA *n* = 61; NF sgRNA + Na_v_1.6-GFP *n* = 28; **p = 0.0099, Student’s *t*-test). **(C,F)** Distance from the soma to start of AIS for AnkG **(C)** and Na_v_ **(F)** in NF sgRNA (data originally from Figure 4) or NF sgRNA + Na_v_1.6-GFP neurons (**C**: NF sgRNA *n* = 44; NF sgRNA + Na_v_1.6-GFP *n* = 44; asterisks indicate statistical significance from sgRNA Ctl.; NF sgRNA *p = 0.012, NF sgRNA + Nav1.6-GFP **p = 0.0073, Kruskal-Wallis ANOVA followed by Dunn’s post-test with Bonferroni correction. **F**: NF sgRNA *n* = 43; NF sgRNA + Na_v_1.6-GFP *n* = 26). sgRNA Ctl. is displayed as a dashed line for comparison. Error bars indicate mean ± SEM. **(G)** To-scale distribution of AnkG (black) and Na_v_ (red) in NF sgRNA (left) and NF sgRNA + Na_v_1.6-GFP (right) neurons. Color saturation of bars are indicative of average labeling intensity of the population. Dashed black line indicates start of AnkG enrichment in sgRNA control (data originally from Figure 4). Left error bar indicates SEM for distance from the soma, right error bar indicates SEM for length; data taken from **(B,C)** and** (E,F)**.

The loss of NF caused a ~20% depletion of overall AnkG enrichment (Figures 4A,B). Recent studies have demonstrated that AnkG can be stabilized by any proteins that contain an AnkG binding domain (Leterrier et al., 2017). Thus, we sought to restore AnkG levels in NF depleted neurons by overexpressing the most distally enriched Na_v_ isoform at the AIS, Na_v_1.6 (Boiko et al., 2003), to determine if its increased presence would rescue the distal translocation of AnkG. To enable visualization of channel overexpression, we expressed a fluorescent chimera, Na_v_1.6-GFP, which has been previously shown to properly traffic to the AIS (Akin et al., 2015) and to exhibit normal gating kinetics (Gasser et al., 2012). Neurons were transfected with both NF sgRNA or a sgRNA control plasmid and Na_v_1.6-GFP, and immunostained for mCherry, GFP, and AnkG or panNa_v_. Na_v_1.6 overexpression rescued the deficient enrichment levels of AnkG in NF sgRNA neurons (81 ± 3% for NF sgRNA, 112 ± 1% for NF sgRNA + Na_v_1.6-GFP; *n* = 63 and 18, respectively; Figures 5A,B; NF sgRNA originally from Figure 4B) as expected. However, overexpression of Na_v_1.6 did not rescue the distal shift of AnkG enrichment we previously observed in NF sgRNA neurons (5.0 ± 1.0 μm for sgRNA Ctl., 8.3 ± 1.2 μm for NF sgRNA, 8.8 ± 1.5 μm for NF sgRNA and Na_v_1.6-GFP; *n* = 52, 44, and 44, respectively; Figure 5C; sgRNA Ctl. and NF sgRNA originally from Figure 4C). These experiments also confirm successful AIS targeting of Na_v_1.6 channels in the absence of NF.

Additionally, we investigated the influence of Na_v_1.6 overexpression on total Na_v_ expression at the AIS to determine if their enrichment or location were altered. Despite normal levels of Na_v_ labeling intensity in NF sgRNA only neurons (Figure 4F), overexpression of Na_v_1.6 did significantly increase the total Na_v_ enrichment as detected by a pan-Na_v_ antibody when NF sgRNA was co-transfected with Na_v_1.6-GFP (94 ± 5% for NF sgRNA, 117 ± 6% for NF sgRNA + Na_v_1.6-GFP; *n* = 61 and 28, respectively; Figures 5D,E; NF sgRNA originally from Figure 4F), further supporting the proper localization of this channel. Additionally, Na_v_ overexpression had no influence on the start of Na_v_ enrichment at the AIS (5.9 ± 0.9 μm for sgRNA Ctl., 7.5 ± 1.7 μm for NF sgRNA, 7.6 ± 1.3 μm for NF sgRNA and Na_v_1.6-GFP; *n* = 62, 43, and 26, respectively; Figure 5F; sgRNA Ctl. and NF sgRNA originally from Figure 4G), demonstrating that the loss of AnkG enrichment is not an indirect effect of other altered binding partners, but is specific to the loss of NF (summary in Figure 5G).

### NF Knockout Neurons Do Not Exhibit Altered Action Potential Initiation, but Generate Wider Action Potentials

**Figure 6:**
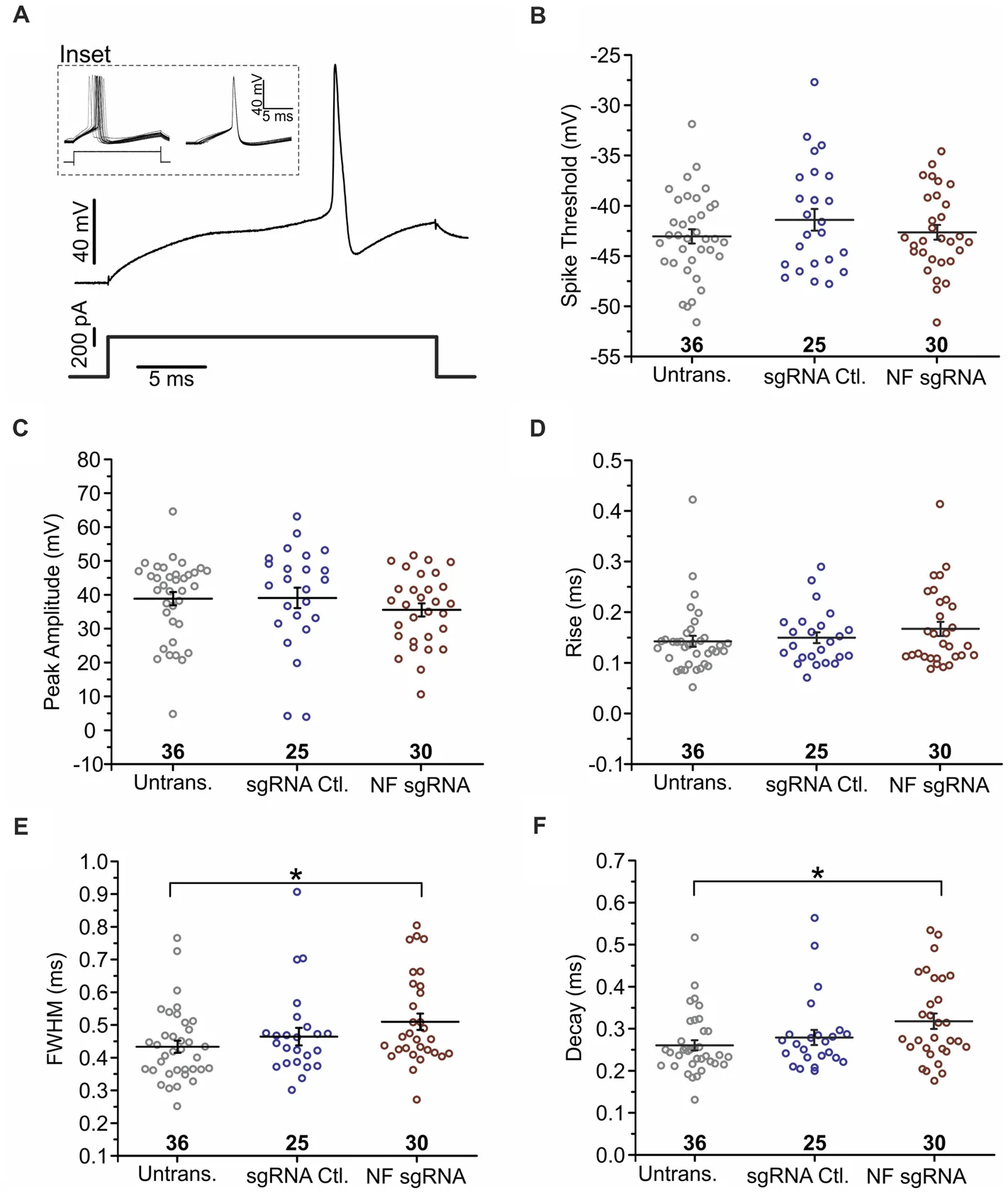
Knockout of NF broadens action potentials. **(A)** Example of a single trace of the voltage change (top) in response to a current step (bottom). Inset shows voltage response to current step for 20 subsequent trials (left), which were then peak aligned (right) and averaged for analysis. **(B)** Measurements of spike threshold (Untransfected *n* = 36; sgRNA Ctl. *n* = 25; NF sgRNA *n* = 30). **(C–F)** Measurements of waveform parameters including peak amplitude (**C**: Untransfected *n* = 36; sgRNA Ctl. *n* = 25; NF sgRNA *n* = 30), rise time (**D**: Untransfected *n* = 36; sgRNA Ctl. *n* = 25; NF sgRNA *n* = 30), full-width at half maximum amplitude (FWHM; **E**: Untransfected *n* = 36; sgRNA Ctl. *n* = 25; NF sgRNA *n* = 30; *p < 0.05, ANOVA with Tukey’s *post hoc* comparisons), and decay time (**F**: Untransfected *n* = 36; sgRNA Ctl. *n* = 25; NF sgRNA *n* = 30; *p < 0.05, ANOVA with Tukey’s *post hoc* comparisons). Error bars indicate mean ± SEM.

Although we found no intensity or localization changes in Na_v_ at the AIS of NF sgRNA neurons, functional differences may be present if NF has an influence directly or indirectly (*via* Na_v_ β subunits) on the kinetics of Na_v_ α subunits (Brackenbury and Isom, 2011). Thus, we investigated the electrical properties of control and NF sgRNA neurons using whole-cell patch clamp electrophysiology. Neurons were transfected with either NF sgRNA or a sgRNA control plasmid and recordings were performed on DIV14–18 in untransfected, NF sgRNA, and sgRNA control neurons. No differences in baseline physiological properties, including resting membrane potential (RMP) and input resistance, were observed across conditions (Table 1). If NF was in fact influencing Na_v_ gating kinetics, one might expect to observe changes in spike threshold (Platkiewicz and Brette, 2011). Thus, we induced action potential generation through just-suprathreshold current injections to closely examine the kinetic parameters of the action potential waveform in each condition (Figure 6A). No significant changes in the spike threshold were observed across conditions (−43.0 ± 0.7 mV for untransfected, −41.4 ± 1.1 mV for sgRNA Ctl., −42.6 ± 0.7 mV for NF sgRNA; *n* = 36, 25, and 30, respectively; Figure 6B). Peak amplitude and rise time, both of which are also indicative of Na_v_ kinetics, also remained unchanged in NF sgRNA neurons (Peak amplitude: 38.9 ± 2.0 mV for untransfected, 39.1 ± 3.0 mV for sgRNA Ctl., 35.5 ± 1.9 mV for NF sgRNA; *n* = 36, 25, and 30, respectively; Figure 6C. Rise time: 0.14 ± 0.01 ms for untransfected, 0.15 ± 0.01 ms for sgRNA Ctl., 0.17 ± 0.01 ms for NF sgRNA; *n* = 36, 25, and 30, respectively; Figure 6D). There was, however, a significant slowing of the action potential between untransfected and NF sgRNA neurons as measured by FWHM (0.44 ± 0.02 ms for untransfected, 0.47 ± 0.03 ms for sgRNA Ctl., 0.51 ± 0.03 ms for NF sgRNA; *n* = 36, 25, and 30, respectively; Figure 6E) and decay time (0.26 ± 0.01 ms for untransfected, 0.28 ± 0.02 ms for sgRNA Ctl., 0.32 ± 0.02 ms for NF sgRNA; *n* = 36, 25, and 30, respectively; Figure 6F). All electrophysiological properties of the neurons recorded in the three groups are summarized in Table 1. These data indicate that Na_v_ gating kinetics are likely unaltered in NF sgRNA neurons, but that other changes result in the widening of action potentials and the increase in decay time. Phase plots of the action potentials (Supplementary Figure S3) demonstrate that the overall threshold for firing (arrow in Supplementary Figure S3B) is similar and the rising and polarizing phases are largely unchanged between conditions.

**Table 1:** Summary of electrophysiological measurements.

	Untransfected	sgRNA Ctl.	NF sgRNA
RMP (mV)	−72.0 ± 0.9	−71.9 ± 1.3	−72.8 ± 1.4
Input resistance (MOhm)	275 ± 20	298 ± 21	236 ± 17
Spike threshold (mV)	−43.0 ± 0.7	−41.4 ± 1.1	−42.6 ± 0.7
Peak amplitude (mV)	38.9 ± 2.0	39.1 ± 3.0	35.5 ± 1.9
Rise time (ms)	0.14 ± 0.01	0.15 ± 0.01	0.17 ± 0.01
FWHM (ms)	0.44 ± 0.02	0.47 ± 0.03	0.51 ± 0.03
Decay time (ms)	0.26 ± 0.01	0.28 ± 0.02	0.32 ± 0.02

*Properties of resting membrane potential (RMP), input resistance, spike threshold, peak amplitude, rise time, full width at half maximum amplitude (FWHM), and decay time are shown. All recordings were made between days in vitro (DIV) 14–18 for untransfected (*n* = 36), single guide RNA (sgRNA) Ctl. (*n* = 25), and neurofascin-186 (NF) sgRNA (*n* = 30) cells. Transfections for sgRNA Ctl. and NF sgRNA were identified with an mCherry marker*.

### Knockout of NF Does Not Influence NrCAM Enrichment or Relative Localization at the AIS

**Figure 7:**
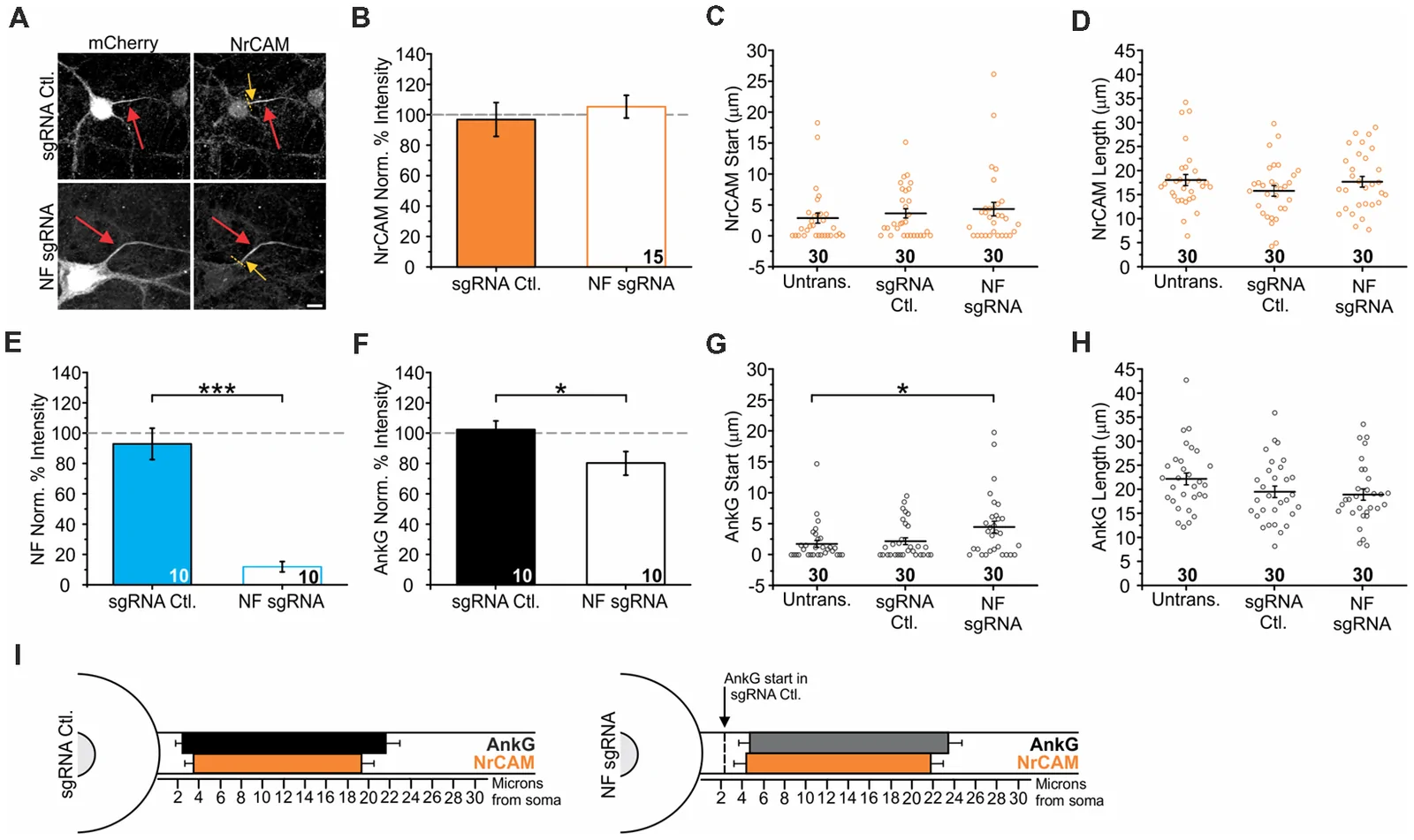
Knockout of NF does not influence neuronal cell adhesion molecule (NrCAM) enrichment or relative localization. **(A)** Representative images of sgRNA control or NF sgRNA stained for mCherry with NrCAM. Red arrows indicate the AIS of transfected neurons; yellow arrows indicate start of AnkG enrichment; yellow dashed lines indicate junction of soma and axon. Scale bar: 10 μm. **(B,F)** Ratio of mean fluorescence intensity of NrCAM** (B)** and AnkG **(F)** at the AIS in the same sgRNA control or NF sgRNA neurons, normalized to untransfected neurons in the same image (**B**: *n* = 15 in both conditions. **F**: *n* = 15 for both conditions; *p = 0.034, Student’s *t*-test). **(C,G)** Distance from the soma to start of AIS for NrCAM **(C)** and AnkG **(G)** in the same untransfected, sgRNA control, and NF sgRNA neurons (**C**: *n* = 30 for all conditions. **G**: *n* = 30 for all conditions; *p = 0.05, Kruskal-Wallis ANOVA followed by Dunn’s post-test with Bonferroni correction). **(D,H)** AIS length for NrCAM **(D)** and AnkG **(H)** in the same untransfected, sgRNA control, and NF sgRNA neurons (**D**: *n* = 30 in all conditions. **H**: *n* = 30 in all conditions). **(E)** Ratio of mean fluorescence intensity of NF at the AIS in paired sgRNA control or NF sgRNA expressing neurons from the same culture as NrCAM data was taken, normalized to untransfected neurons in the same image (*n* = 10 in both conditions; ***p < 0.001, Student’s *t*-test). Error bars indicate mean ± SEM. **(I)** To-scale distribution of AnkG (black) and NrCAM (orange) in sgRNA control (left) and NF sgRNA (right) neurons. Color saturation of bars are indicative of average labeling intensity of the population. Dashed black line indicates start of AnkG enrichment in sgRNA control. Left error bar indicates SEM for distance from the soma, right error bar indicates SEM for length; data taken from **(B–D)** and **(F–H)**.

Previous studies in Purkinje neurons using a NF knockout mouse reported impaired action potential generation as well as a loss of NrCAM localization at the AIS (Zonta et al., 2011). However, using shRNA in hippocampal neurons produces only a very moderate effect on NrCAM accumulation (Hedstrom et al., 2007). Given the modest functional effects of NF sgRNA on action potential firing in our neurons, we next investigated how the loss of NF might alter NrCAM enrichment at the AIS using more efficient depletion methods. Neurons were transfected with NF sgRNA or a sgRNA control plasmid and fixed and immunostained for mCherry, NrCAM, and AnkG or NF. In NF sgRNA neurons, there was no decrease in NrCAM intensity compared to the sgRNA control (97 ± 11% for sgRNA Ctl. and 105 ± 8% for NF sgRNA; *n* = 15 for both conditions; Figures 7A,B). NrCAM’s AIS localization index was also statistically indistinguishable from AnkG (Supplementary Figure S2D). Moreover, there were no changes in the AIS start (2.8 ± 0.8 μm for untransfected, 3.5 ± 0.7 μm for sgRNA Ctl., and 4.2 ± 1.1 μm for NF sgRNA; *n* = 30 for all conditions; Figure 7C) or length (18.0 ± 1.2 μm for untransfected, 15.7 ± 1.1 μm for sgRNA Ctl., and 17.6 ± 1.1 μm for NF sgRNA; *n* = 30 for all conditions; Figure 7D) as measured through NrCAM labeling. To ensure that the NF sgRNA construct was still efficient in our NrCAM measurements, a subset of neurons from the same culture transfected with either the sgRNA control or NF sgRNA was stained for NF and quantified for enrichment levels. These neurons also exhibited a significant decrease in NF labeling intensity, similar to that shown in Figure 3 (93 ± 10% for sgRNA Ctl. and 12 ± 3% for NF sgRNA; *n* = 10 for both conditions; Figure 7E). Likewise, measuring AnkG staining in the same neurons as NrCAM was quantified and also confirmed NF-dependent modulation of AnkG enrichment levels and localization (Figures 7F–H, **F**: 102 ± 6% for sgRNA Ctl. and 80 ± 8% for NF sgRNA; *n* = 10 for both conditions; **G**: 1.8 ± 0.6 μm for untransfected, 2.2 ± 0.6 μm for sgRNA Ctl., and 4.5 ± 0.9 μm for NF sgRNA; *n* = 30 for all conditions; **H**: 22.1 ± 1.2 μm for untransfected, 19.5 ± 1.2 μm for sgRNA Ctl., and 18.9 ± 1.2 μm for NF sgRNA; *n* = 30 for all conditions). Together, these results indicate that NrCAM expression is undisturbed by NF depletion and, like Na_v_, display a relative localization uncoupled from AnkG (summary in Figure 7I).

## Discussion

The AIS has been an area of interest for understanding neuronal excitability for nearly 50 years. This unique neural compartment has been extensively studied, and while the primary molecular components have been identified, their interplay is yet to be completely explained. Multiple studies agree that AnkG is a master regulator of this structure, necessary for both its formation and maintenance (Zhou et al., 1998; Zhang and Rasband, 2016). AnkG also interacts with the majority of other AIS proteins (Na_v_, NF, βIV-spectrin), further confirming its critical role in AIS organization (Xu et al., 2013; Leterrier et al., 2015). These interactions are typically thought to create a tightly-linked structure with slow turnover and negligible diffusion (Hedstrom et al., 2008; Akin et al., 2015). Here, we describe a disruption in AnkG accumulation as well as an uncoupling of AnkG localization relative to Na_v_ and NrCAM caused by the acute loss of NF (Figure 4B). These data agree with a recently published study showing more promiscuous interactions of proteins containing AnkG binding motifs that ensure a stable “interactome” for enrichment of AIS components (Leterrier et al., 2017). However, the relative arrangement of proteins within the AIS seems quite specific, as the selective depletion of NF causes a distal shift in AnkG localization without changing the location of Na_v_ (Figures 4C,G) or NrCAM (Figure 7C). Overexpression of Na_v_1.6 was unable to restore proximal relative localization of AnkG when NF was depleted despite restoring overall levels of AnkG (Figures 5B,C). Moreover, given no functional changes in Na_v_ as a result of altered AnkG localization (Figures 6B–D), NF does not appear to directly influence cellular excitability through modulation of Na_v_ channels. These results suggest that the anchoring of Na_v_ is not solely controlled by AnkG and that NF plays a role in stabilization of the mature AIS but does not directly alter Na_v_ kinetics.

Although NF can bind to both Na_v_ and AnkG, its loss influences them differently. The knockout of NF had a two-fold effect on AnkG, altering its concentration at the AIS and shifting its overall localization within the AIS (Figure 4). Typically, depletion of either AnkG or Na_v_ leads to a loss of the other and eventually a destabilization of the AIS as whole (Zhou et al., 1998; Xu and Shrager, 2005). Conversely, without NF the two proteins act independently. Indeed, we now appreciate that the AIS is not a static structure, as several studies have observed alterations in AIS morphology through manipulations of neural input (Grubb and Burrone, 2010a; Kuba et al., 2010; Evans et al., 2015). This plasticity occurs through shifts in AIS location relative to the soma (Grubb and Burrone, 2010a) or alterations in AIS length (Kuba et al., 2010; Evans et al., 2015). In all cases, Na_v_ and AnkG have been shown to transform in tandem, maintaining their relative proximal and distal enrichment patterns (along with NF and β-spectrin). Most studies of AIS structural plasticity have only shown that dynamic relocations occur during very early development or *in vitro* (Yamada and Kuba, 2016), a limitation of this study as well. It would be increasingly exciting to study AIS plasticity *in vivo* (Jamann et al., 2018). We propose that the coupling of AnkG to Na_v_ and NrCAM minimally requires a contribution from NF. Therefore, NF may be one of multiple players required for specific interactions that stabilize the AIS.

There are conflicting data regarding the exact role of NF from studies of Purkinje neurons in NF knockout mice and those using RNA interference in hippocampal or cortical neurons. Purkinje neurons in knockout mice have fairly normal AIS development, including AnkG and Na_v_ enrichment, with only a loss of NrCAM. However, during maturation, NF knockout leads to a complete dismantling of the AIS (Zonta et al., 2011). Using shRNA to deplete NF in hippocampal neurons had a much milder phenotype, where the AIS still recruits Na_v_ and AnkG (Hedstrom et al., 2007), though complete enrichment of AnkG is impaired (Leterrier et al., 2017). This differs from studies at the nodes of Ranvier, where NF is crucial to recruiting Na_v_ (Sherman et al., 2005). Using a CRISPR strategy to more thoroughly deplete NF from the AIS, we observed a significant loss of AnkG accumulation that did not lead to the disassembly of the mature AIS (Figure 4), in agreement with previous knockdown experiments (Leterrier et al., 2017). Additionally, we did not see the disruption of NrCAM enrichment (Figure 7), which was observed in cultured slices of Purkinje neurons (Zonta et al., 2011). We speculate that there are at least three possibilities for these discrepancies between our study and others. First, there are differences in the temporal windows over which manipulations to NF were applied. We do not, however, believe this to be the cause since even in null mice initial AnkG localization and Na_v_ recruitment were normal. Second, there are differences between the firing frequencies of the neurons studied. The firing rates of Purkinje (>100 Hz) and hippocampal (<10 Hz) neurons differ by an order of magnitude. We do not believe this is the cause of instability, as while the loss of NF decreased excitability in Purkinje neurons (Zonta et al., 2011), decreasing excitability is not usually detrimental to the stability of the AIS. Interestingly, we did detect some changes in width and decay time of the somatically-recorded action potential waveform, which may indicate that NF influences K^+^ channels in the AIS. That being said, the phase plots (Supplementary Figure S2) and overall properties of the cells (Table 1) were not telling as to a mechanism. These results could be due to other compensatory mechanisms. In our study we only compared the firing of single action potentials, but perhaps a slight increase in action potential width and decay time as we observed could indirectly impair the ability of neurons to maintain high frequency firing as seen in Purkinje neurons (Zonta et al., 2011), especially given the different cell shape and physiology. Third, there are additional isoforms of NF present in our cultures that were not targeted by our sgRNA. There is the glial isoform, NF-155, as well as the more recently discovered NF-140 (Zhang et al., 2015). While in null mice the glial NF-155 is also ablated (Sherman et al., 2005), these isoforms remain in our cultures. However, the selective knockout of NF-155 produces independent impairments (Smigiel et al., 2018), arguing against potentially redundant transcellular signaling by NF-155 within the extracellular matrix (ECM). Furthermore, NF-140 has remained understudied until recently. This isoform has been shown to be expressed in the AIS of cerebellar Purkinkje neurons in a developmental manner, and while it plays complementary roles in Na_v_ and NrCAM clustering (Zhang et al., 2015), our staining with a pan-NF antibody showing nearly complete lack of staining at the AIS suggests that this isoform is not present at the AIS in our neurons (Figure 3). Thus, we argue that additional factors may be responsible for the more drastic destabilization previously seen in Purkinje neurons (Zonta et al., 2011).

Without the presence of NF during development, the AIS may be lacking critical extracellular interactions. Among these could be interactions with ECM proteins such as brevican. The clustering of brevican and formation of a specialized brevican-containing matrix at the AIS has been shown to be dependent on NF, and has been speculated as important for stabilizing axo-axonic synapses (Hedstrom et al., 2007). Additionally, NF may play a more direct role in the formation of axo-axonic GABAergic synapses along the axon hillock (Kriebel et al., 2011). Coincidently, Purkinje neurons have one of the largest inhibitory inputs onto their AIS from basket cells that form Pinceau synapses in this region. This innervation is dramatically disrupted by the loss of NF (Ango et al., 2004), which may contribute to the more dramatic phenotype observed in those neurons. Moreover, axo-axonic inhibitory synapses are also found in the cortex, hippocampus, and amygdala. These connections appear to be disrupted in schizophrenia (Lewis, 2011), and genetic depletion of NF in mature neurons within the amygdala was recently demonstrated to alter reversal learning in fear-conditioning studies (Saha et al., 2018), further pointing to a highly important role for these connections. Additionally, during acute changes in AIS location in cultured neurons, GABA receptors were destabilized within the AIS (Muir and Kittler, 2014), though their relative location during plasticity-induced relocation of AnkG remained stable (Wefelmeyer et al., 2015). Functional studies of the relatively static inhibitory synapses at the AIS could not be performed, but were modeled and observed to change the relative inhibition of neurons in a homeostatic manner during AIS distal shifts (Wefelmeyer et al., 2015). Given our results that NF helps to couple the localization of both AnkG and Na_v_ within the AIS, this protein is well positioned to influence GABAergic innervation and function within the AIS and could be studied in relation to AnkG and Na_v_ translocation in axons heavily innervated with GABAergic synapses.

After revealing that NF ablation reduces the distal enrichment of AnkG relative to Na_v_, we are left to speculate about the molecular control of this alignment within the AIS. A number of other dynamic factors can alter channel density and availability at the AIS including channel endocytosis (Benned-Jensen et al., 2016), Ca^2+^ influx (Bender et al., 2010; Martinello et al., 2015), intracellular fibroblast growth factor homologous factors (Pablo and Pitt, 2016), and AIS-specific kinases and phosphatases (Bréchet et al., 2008; Hien et al., 2014; Xu and Cooper, 2015; Lezmy et al., 2017). Our work cannot directly link to any of these particular mechanisms. Protein kinase CK2 is highly enriched at the AIS and within the nodes of Ranvier of hippocampal neurons *in vitro* and *in vivo* as is AnkG, Na_v_ and NF. CK2 has been found to regulate the interaction of both Na_v_ and the voltage-gated potassium channel (K_v_) K_v_7 in a potentially competitive manner with AnkG, and is also important in enabling a form of K_v_7-dependent AIS plasticity (Lezmy et al., 2017). Pharmacological inhibition of CK2 causes reduced enrichment of both AnkG and Na_v_ at the AIS suggesting a critical role for the kinase to ensure a stable interaction and enrichment of these two proteins at the AIS. How CK2 is localized within the AIS to control the important interaction between Na_v_, K_v_, and AnkG remains to be determined, but data suggest that the Na_v_ themselves recruit the kinase to the AIS (Hien et al., 2014). We speculate that NF may actually play a role in facilitating this localization between CK2, Na_v_, and AnkG, perhaps at the expense of K_v_7. This may partially explain why AnkG enrichment is selectively reduced without NF because of a mismatch in localization between AnkG and CK2-phosphorylated Na_v_. Future development of genetically encoded fluorescent Na^+^ and K^+^ indicators (Shen et al., 2018) to study channel function within the AIS as well as the continued development of super-resolution microscopy (Sigal et al., 2018) may help investigate this mechanism further.

## Data Availability

All datasets generated for this study are included in the manuscript and the supplementary files.

## Author Contributions

SA: conception and design, experiments and data acquisition, analysis and interpretation of the data, draft and revision of the article. AB: experiments and data acquisition, analysis and interpretation of the data, draft and revision of the article. AG and MH: concept and design, interpretation of data, draft and revision of the article.

## Conflict of Interest Statement

The authors declare that the research was conducted in the absence of any commercial or financial relationships that could be construed as a potential conflict of interest.

## Footnotes

^1^ https://fiji.sc/

^2^ https://www.mathworks.com/products/matlab.html
