# Supplementary material for: Loss of Neurofascin-186 Disrupts Alignment of AnkyrinG Relative to Its Binding Partners in the Axon Initial Segment
Source: Front Cell Neurosci. 2019 Jan 22;13:1. doi: 10.3389/fncel.2019.00001 (PMC6349729; doi:10.3389/fncel.2019.00001)
Supplement: Supplementary file 1 [file Data_Sheet_1.docx]

Supplementary Material

Loss of Neurofascin-186 Disrupts Alignment of AnkyrinG Relative to its Binding Partners in the Axon Initial Segment

Scott Alpizar, Arielle Baker, Allan Gulledge, Michael Hoppa^*^

*** Correspondence:** Michael Hoppa (Michael.B.Hoppa@dartmouth.edu)

# Supplementary Figures


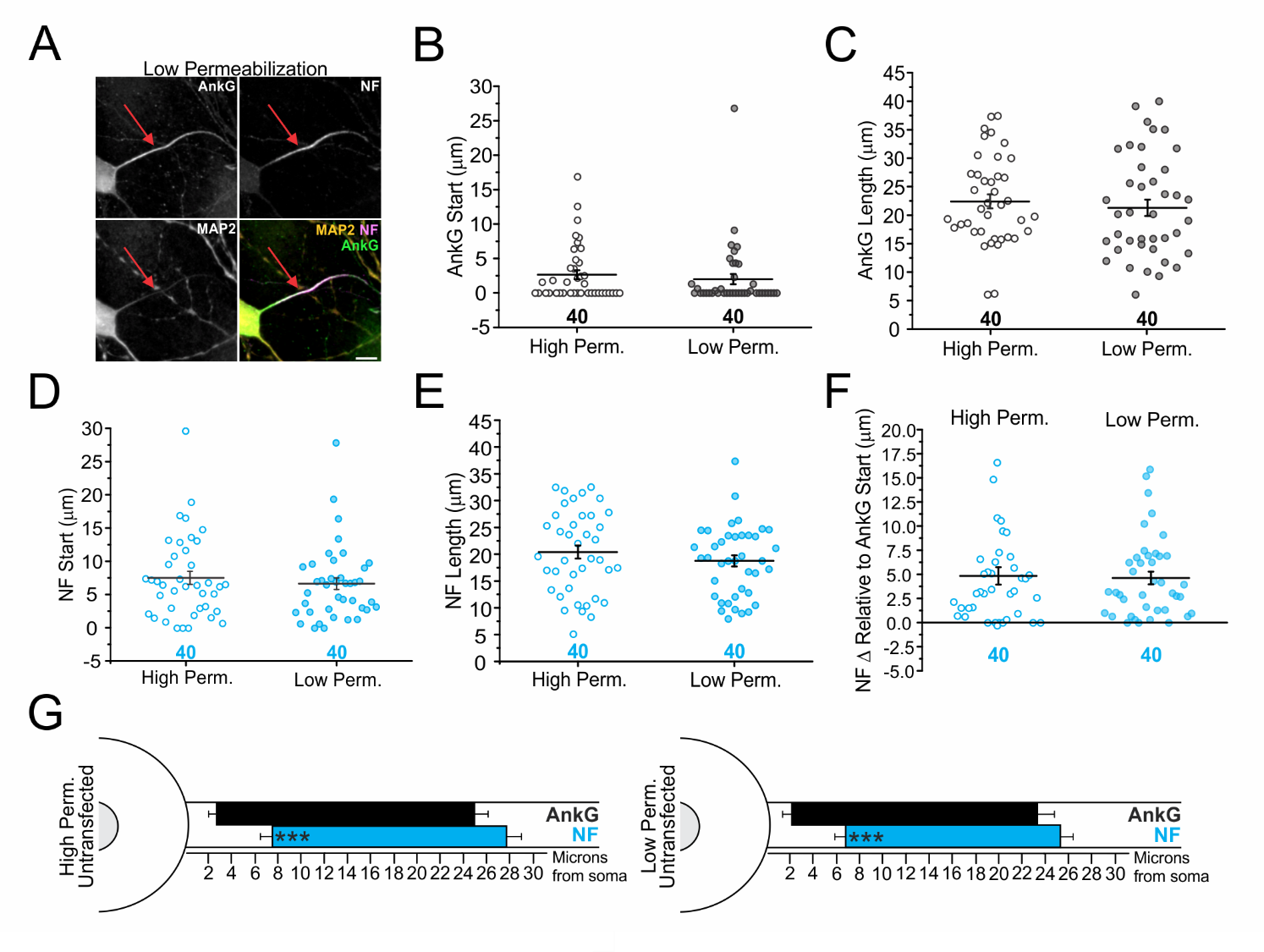


## Supplementary Figure 1. Permeabilization conditions do not alter AIS immunostaining of AnkG and NF.

**(A)** Representative images of immunostaining for AnkG, NF and MAP2 in cells permeabilized by 0.2% triton. Red arrows indicate the AIS. Scale bar: 10 µm. **(B-C)** Distance from soma to start of AnkG enrichment **(B)** and length **(C)** of AnkG in neurons permeabilized by 10% Triton X-100 (High Perm.; open circles) or 0.2% Triton X-100 (Low Perm.; filled circles). **(D-E)** Distance from soma to start of AnkG enrichment **(D)** and length **(E)** of NF in neurons permeabilized by 10% Triton X-100 (High Perm.; open circles) or 0.2% Triton X-100 (Low Perm.; filled circles). **(F)** Comparison of start of NF protein enrichment relative to start of AnkG enrichment in both 10% Triton X-100 (High Perm.; open circles) and 0.2% Triton X-100 (Low Perm.; filled circles). Error bars indicate mean ± SEM, n = 40 for all conditions. **(G)** To-scale distribution of AnkG (black) and NF (blue) in 10% Triton X-100 (High Perm.; left) and 0.2% Triton X-100 Low Perm.; right) neurons. Asterisks indicate significance between the AIS start of NF and AnkG within each permeabilization condition; ****p* < 0.001 for High Perm. and ***p < 0.001 for Low Perm., Wilcoxon Signed-Ranks test. Left error bar indicates SEM for distance from soma, right error bar indicates SEM for length; data taken from panels **(B-E)**.


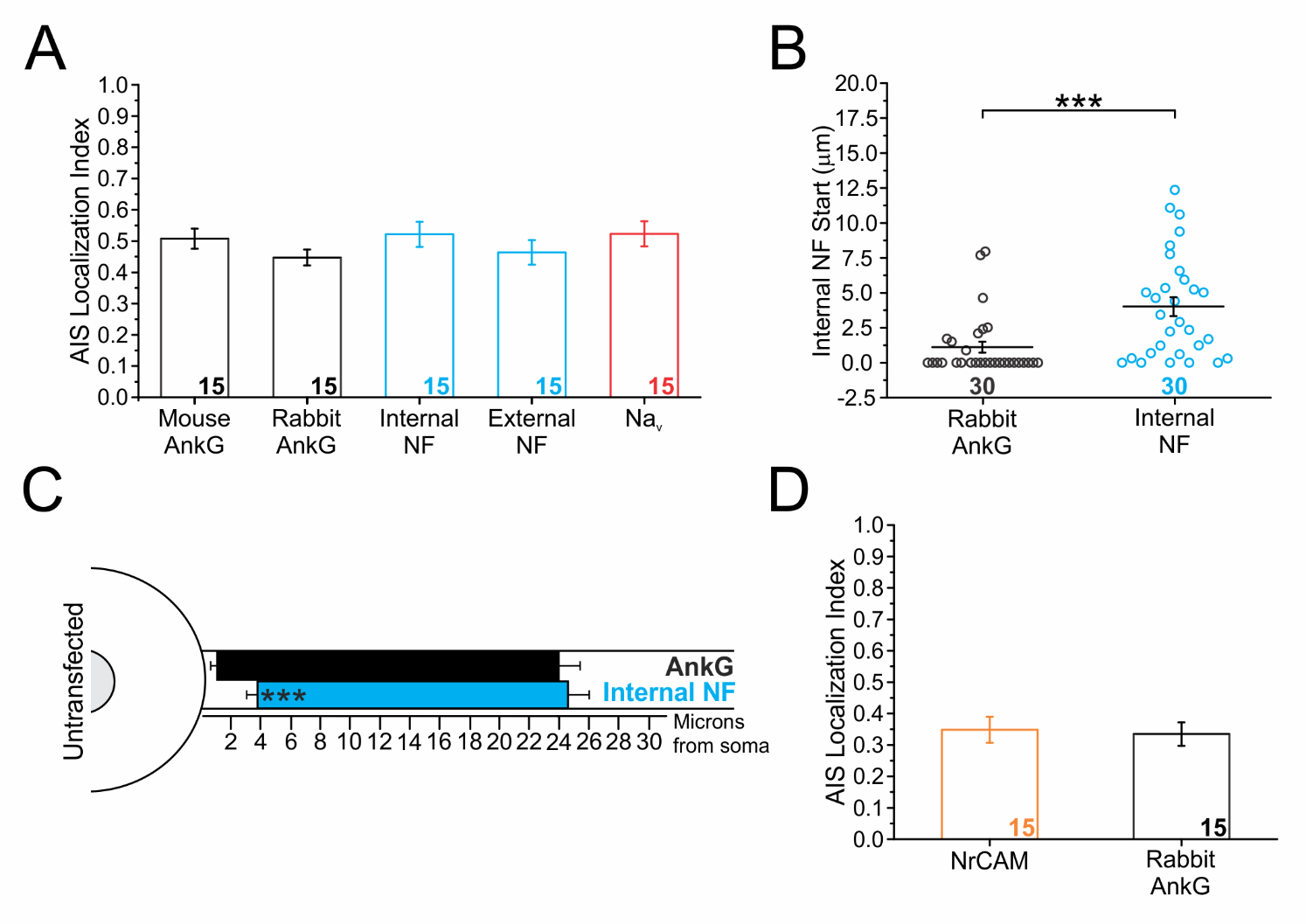


**Supplementary Figure 2. Antibody combinations do not alter AIS immunostaining.**

**(A)** AIS localization index values for AnkG, NF, and Na_v_ immunostaining detected using a wide-field microscope. **(B)** Distance from soma to start of AnkG (black) and NF (cyan) enrichment in untransfected neurons using a rabbit AnkG antibody as well an antibody directed against the intracellular domain of NF. Error bars indicate mean ± SEM (n = 30 for both proteins; ****p* < 0.001, Wilcoxon Signed-Ranks test). **(C)** To-scale distribution of AnkG (black) and NF (blue) in untransfected neurons. Asterisks indicate significance between the AIS start of NF and AnkG. Left error bar indicates SEM for distance from soma, right error bar indicates SEM for length; data taken from panel **(B)**. **(D)** AIS localization index values for AnkG (rabbit) and NrCAM immunostaining detected using a confocal microscope.


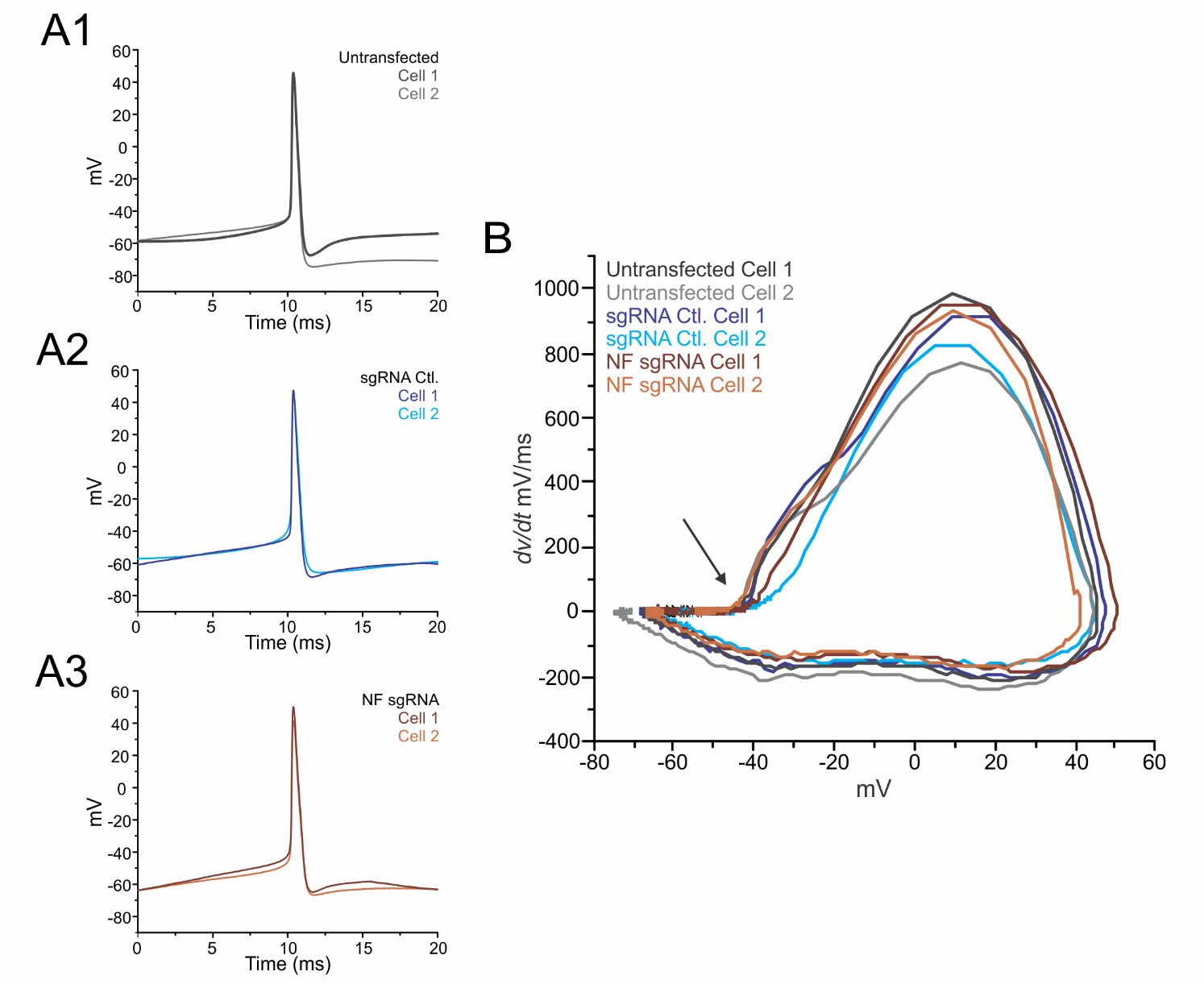


**Supplementary Figure 3.** **Action potential waveforms kinetics are minimally altered by the loss of NF.**

**(A1-3)** Representative traces of recorded action potentials from two cells in each untransfected (**A1**), sgRNA Ctl. (**A2**), and NF sgRNA (**A3**) conditions. **(B)** Overlaid phase plots of the action potential waveforms for all action potentials shown in **(A)** with corresponding colors. Arrow indicates the inflection that represents the firing threshold.
